# Patellofemoral pain syndrome in children and adolescents: A cross-sectional study

**DOI:** 10.1371/journal.pone.0300683

**Published:** 2024-04-16

**Authors:** Geronimo José Bouzas Sanchis, Joubert Vitor de Souto Barbosa, Rafael Limeira Cavalcanti, Josiane Pereira Bezerra, Maristela Linhares dos Santos, Thais Sousa Rodrigues Guedes, Sanderson José Costa de Assis, Rebeca de Castro Santana, Johnnatas Mikael Lopes, Angelo Giuseppe Roncalli da Costa Oliveira, Marcello Barbosa Otoni Gonçalves Guedes

**Affiliations:** 1 Department of Physiotherapy, Federal University of Minas Gerais, Belo Horizonte, MG, Brazil; 2 Department of Physiotherapy, University of Valencia, Buenos Aires, Valencia, Spain; 3 Department of Physiotherapy, Postgraduate Program in Physiotherapy, Federal University of Rio Grande do Norte, UFRN, Natal, RN, Brazil; 4 Department of Physiotherapy, Federal University of Rio Grande do Norte, UFRN, Natal, RN, Brazil; 5 Department of Dentistry, Postgraduate Program in Public health, Federal University of Rio Grande do Norte, UFRN, Natal, RN, Brazil; 6 Department of Physiotherapy, Postgraduate Program in Public health, Federal University of Rio Grande do Norte, UFRN, Natal, RN, Brazil; 7 Department of Physiotherapy, Federal University of São Francisco Valley, Paulo Afonso, BA, Brazil; Ningbo University, CHINA

## Abstract

**Objectives:**

To assess the prevalence and associated factors of Patellofemoral Pain Syndrome (PFPS) in children and adolescents.

**Method:**

A population-based cross-sectional study was conducted with children and adolescents aged 10 to 18 years, who presented a history of peripatellar and/or retropatellar pain, attending elementary or high school in urban public schools in Natal, Brazil. The sample size was calculated based on a minimum outcome prevalence of 22%.

**Results:**

A prevalence of 24.7% of PFPS was found. There was a positive association of PFPS with active students (p < 0.01; PR: 2.5; CI: 1.4–4.5), low functional capacity (p < 0.01; PR: 8.0; CI: 5.0–12.8), and those classified as pubertal (p < 0.03; PR: 1.8; CI: 1.0–3.2).

**Conclusion:**

There was a considerable prevalence of PFPS in children and adolescents, as well as an association between the level of sexual maturation and adjustable determinants, such as the level of physical activity and low functional capacity in this group.

## Introduction

Patellofemoral Pain Syndrome (PFPS) is one of the most commonly observed painful dysfunctions in the knee joint, primarily affecting adolescents and young adults, predominantly females [1]. Its prevalence in the global population varies between 22.7% and 28.9% [2], especially in individuals in the age range of 12 to 19 years [3]. In the national context, the available scientific literature on this topic is still limited, making it difficult to determine the exact prevalence of PFPS in the child and adolescent population in Brazil [4].

Chronic pain caused by PFPS has a significant impact on the lives of this population, leading to functional limitations and adversely affecting their daily activities as well as their social participation [5, 6]. Moreover, it is estimated that about 12% of primary care medical visits are made by individuals with PFPS [5, 7]. Despite the increasing demand for medical attention in recent years, uncertainties persist regarding the factors associated with PFPS in children and adolescents [5, 7].

Due to its multifactorial nature, PFPS has been associated with one or more extrinsic and intrinsic elements [8], such as biomechanical and postural variations [9], imbalances in stabilizing muscle groups [9], proprioceptive changes in the ankle-foot [9], mechanisms related to sexual maturation [10], levels of physical activity, and functional capacity [6].

Markers of the sexual maturation period, for instance, play a significant role during childhood and adolescence [11]. Therefore, PFPS is one of the most common musculoskeletal disorders during this developmental period, standing out among knee pain dysfunctions in young people and children [3]. Furthermore, it is reasonable to consider that the level of physical activity and individual characteristics can influence the development of PFPS, as around 25% of knee injuries related to physical activity are attributed to this dysfunction [12]. Pain reports are common in individuals affected by PFPS and can limit the performance of physical and functional activities, thus reducing their social participation [4].

Current evidence focuses on specific populations of adolescents, such as young athletes, without exploring the prevalence of PFPS in the general young population [4]. Investigating variables associated with PFPS in young people is relevant for the development of public policies, prevention, and appropriate interventions aimed at reducing the negative impact of the condition in their lives. In this context, the objective of this study is to assess the prevalence of PFPS and its association with intrinsic and extrinsic factors in students between 10 and 18 years of age. We hope that these findings will provide a better understanding of the influence of these variables on the occurrence of PFPS in Brazilian students.

## Methods

### Study design

This was a cross-sectional, population-based study that aimed to estimate the prevalence of Patellofemoral Pain Syndrome (PFPS) and investigate associated factors. The sample size was calculated based on a minimum prevalence of 22%, according to data from Saes & Soares [4] (2017). However, to account for selection bias, loss to follow-up, and systematic errors, the sample size was increased by 5%, totaling 277 individuals to be evaluated. The data collection commenced in October 2019 and was interrupted in March 2020 (due to the COVID-19 pandemic). It was resumed in February 2022 and concluded in November of the same year. The project was approved by the Ethics Committee on Research (CEP) of Onofre Lopes University Hospital (CAAE: 07389318.1.0000.5292), and all participants and their legal guardians signed an informed consent form.

Thirty schools were selected out of a total of 200 using the cluster sampling technique, based on Probability Proportional to Size (PPS). Subsequently, students were selected using systematic random sampling, with a sampling interval 28.5, from the selected schools.

According to eligibility criteria, the study included children and adolescents aged 10 to 18 years, enrolled in urban public schools in the city of Natal, Brazil, during the morning or afternoon. The study employed the diagnostic criteria outlined by Crossley et al. (2016). According to these criteria, the primary defining characteristic of patellofemoral pain syndrome is the presence of pain around or behind the patella, exacerbated or elicited by at least one activity that places a load on the femoropatellar joint while bearing weight on the flexed knee (e.g., squatting, ascending, and descending stairs, running, jumping, prolonged sitting) [5]. To refine the selection criteria, the authors specified that a positive classification for the patellofemoral group required the presented symptoms to persist for at least one month, with peripatellar or retropatellar pain worsening during at least two of the aforementioned activities [5]. Children and adolescents with physical and/or mental disabilities or conditions preventing them from maintaining an upright position were excluded from the research.

### Data collection procedures

The assessment was conducted by a team of two healthcare program students and a physiotherapist with five years of clinical practice experience. The students received prior training in questionnaire administration. The assessment took approximately 30 minutes and was conducted at a single time point, with students barefoot and in their school attire. The following outcomes were assessed during the evaluation: Level of physical activity, Posterior chain flexibility, Sexual maturation, and BMI. For the assessment items, the questionnaires, general data, and weight/height were collected by all three researchers.

To assess the level of physical activity, the short version of the International Physical Activity Questionnaire (IPAQ), translated and validated for use in Brazil, was administered. This questionnaire considers activities practiced for at least ten continuous minutes during the previous week, based on self-reports of frequency, intensity, and duration, classifying individuals as very active, active, irregularly active A or B, or sedentary [13]. For the classification, sedentary individuals are those who did not engage in any physical activity for at least 10 continuous minutes in the past week. Irregularly active-A individuals engage in activities on five or more days per week or for 150 minutes per week. Irregularly active-B individuals do not meet the criteria for recommendation A in terms of frequency and duration. Active individuals engage in moderate or vigorous activities on three or more days per week, with sessions lasting at least 20 minutes each, or moderate physical activity or walking on five or more days per week, with sessions lasting at least 30 minutes each [13]. For analysis purposes, the categories Sedentary, irregularly active A, and B were combined into a single category: Irregularly active. The categories Active and Very Active remained identical to the original classification.

Sexual maturation was assessed using the Tanner self-assessment test of pubic hair growth, which has shown satisfactory agreement with medical assessment and is effective in determining the maturation stage in both males and females [14]. The individual undergoing the test was provided with an explanation of the procedure, emphasizing the importance of reliable results. The individual was then taken to a private area where pictures displaying different stages of pubic hair development (ranging from 1 to 5) were displayed, and they identified the picture that most closely resembled their current maturation stage. For statistical analysis, the variables were divided into two groups: pubertal (stages 1 to 4) and post-pubertal (stage 5).

To assess posterior chain flexibility, the student, with knees extended, slowly lowered their head and torso, reaching towards the floor without forcing the movement [15]. The evaluator, observing from the front, measured the hand-floor distance. To quantify this variable, the student had to touch the floor with their fingertips, and if they couldn’t, the distance in centimeters between the tip of the extended middle finger and the floor was measured using a measuring tape. For statistical analysis, individuals who could touch the floor were classified as having adequate flexibility, while those who couldn’t were classified as having inadequate flexibility [15].

The anthropometric variables assessed were body weight, height, and Body Mass Index (BMI). Body weight was measured using a digital scale (Filizola) with a precision of 100g. The procedure was conducted with the student barefoot and in school attire (pants and shorts) in a restricted area of the school [16]. Subsequently, height was measured using a standing stadiometer with centimeter graduation and 1mm precision, fixed to the wall. Height was measured with the individual in an upright position, with bare feet together, head in the horizontal plane at the end of a deep breath. BMI was then calculated by considering the ratio of body weight to the square of height (kg/m2) and classified according to age- and gender-specific reference values suggested by the National Centre for Health Statistics [16].

Functional impairment was assessed using the Portuguese version of the Anterior Knee Pain Scale–Kujala Questionnaire. The questionnaire is used to assess subjective symptoms such as anterior knee pain and functional limitations [17]. The questionnaire items assessed claudication, pain, walking, stair climbing, and sitting for extended periods with flexed knees. It is scored on a scale from 0 to 100, where 100 represents no pain and functional limitations, and 0 indicates constant pain and multiple functional limitations [18]. To categorize the variable, the lower percentile was used; thus, individuals with a score of ≥ 84 in the final score were classified as having a functional impairment.

### Data analysis

Demographic characteristics, gender, and age were described through tabulations, considering the complex sampling design. The prevalence of each variable was calculated based on the complex sampling design, including weights and clustering effects [19]. To analyze the association between PFPS and each independent variable (gender, physical activity, BMI, sexual maturation, posterior chain flexibility, and functional impairment), Prevalence Ratios (PR) and their respective 95% confidence intervals for the outcome were estimated using Multiple Poisson Regression with robust variance. A significance level of 5% (α < 0.05) was adopted. SPSS (version 25) was used for the analysis of demographic characteristics and PR, and Stata (Stata/IC 12.0) was used for Multiple Poisson Regression.

## Results

The study involved the participation of 283 young students, of which: 50.9% (CI: 43.6–58.1) were female, 24.7% (CI: 19–31.4) had PFPS, 58.1% (CI: 50.5–65.3) were aged between 16 and 18 years, 45.9% (CI: 38.5–53.3) were classified as physically active, 27% (CI: 20.6–34.7) had overweight BMI, 70.3% (CI: 63.2–76.5) were in the pubertal phase, and 77.7% (CI: 52.5–86.7) did not exhibit functional impairment. The characterization of the study participants is detailed in the S1 Table.

When analyzing the independent variables, we observed a positive association both in the unadjusted model (p <0.01; PR: 2.5; CI: 1.3–4.4) and in the adjusted model (p <0.01; PR: 2.5; CI: 1.4–4.5) for individuals classified as physically active according to the IPAQ. Regarding sexual maturation, pubertal individuals showed a positive association both in the unadjusted model (p <0.02; PR: 1.9; CI: 1.1–3.3) and in the adjusted model (p = 0.03; PR: 1.8; CI: 1.0–3.2); they also showed an association with functional impairment both in the unadjusted model (p<0.01; PR: 8.2; CI: 5.3–12.2) and in the adjusted model (p<0.01; PR: 8.0; CI: 5.0–12.8). The relationships between PFPS and the independent variables of the study can be seen in the S2 Table.

## Discussion

This study conducted a population-based investigation into the prevalence of PFPS in children and adolescents, aiming to identify possible associations between this painful disorder and various intrinsic and extrinsic factors (level of physical activity, functional capacity, sexual maturation, BMI, and posterior chain flexibility). It is worth noting that there was a positive association of PFPS with young people classified as physically active (IPAQ), pubertal, and with reduced functional capacity, as well as a considerable prevalence of this syndrome in this group.

In relation to the overall prevalence, our findings are like another study that obtained comparable results in an adult population [20], as well as to one that assessed knee pain in young Brazilian students [4]. However, our results differ from another study that found a prevalence of 7.2% in a general population of young individuals [21]. These differences may have been caused by two phenomena: (1) the profile of the population and healthcare systems differing between developing and developed countries, and (2) changes in the diagnostic criteria of SDPF over the years. Regardless of the comparison between other studies, we evaluate our overall prevalence of SDPF as high. Therefore, this condition can be considered a public health problem for health managers to consider when developing public policies for young people and adolescents.

Regarding the prevalence of PFPS by gender, no differences were observed. This finding differs from previous studies that assessed the prevalence of PFPS in the general adult population and did not find this association [20]. However, another study that assessed adolescent athletes of both sexes reported a prevalence of PFPS in 21% of females and 16% of males [22]. A higher prevalence in females may be associated with biomechanical differences, with one of the factors being a decrease in lower limb and trunk muscle strength [9]. The above-mentioned studies were not conducted in Brazil and evaluated specific populations, unlike our study, which assessed the general student population. Thus, based on our findings, planning for the prevention of PFPS risk factors in children and adolescents through public policy development could be directed toward both sexes.

Relating to the level of physical activity, individuals classified as active were positively associated with the study outcome. However, for individuals classified as very active, no significant association was found, which does not corroborate the study by Willy et al. [3], which identified a high level of physical activity as a factor related to PFPS in adolescents and moderate activity as a non-associated factor. In addition, recent studies have suggested that high levels of physical activity in adolescents are associated with a higher risk of PFPS [23]. However, because this is a cross-sectional study, it is possible that pain influenced the physical activity level of individuals classified with PFPS, leading to reduced functional capacity and consequently lower physical activity.

Despite the abundance of research in specific populations, there is a lack of studies focusing on young schoolchildren in developing countries. Our findings support previous studies that high levels of physical activity in adolescents are associated with a higher risk of PFPS. This can be explained by the fact that adolescents who engage in high levels of physical activity are more exposed to high patellofemoral joint stresses, leading to cartilage degradation and contributing to the development and worsening of PFPS [6]. As a result, our study provides valuable insights into this expanding body of knowledge, offering more evidence and a comprehensive understanding of the relationship between the level of physical activity and PFPS.

We also observed a positive association between PFPS and sexual maturation in the pubertal stage. The unique effects of maturation can increase the risk of developing patellofemoral pain [24]. During this period of sexual maturation, adolescents undergo musculoskeletal changes and hormonal imbalances, which, in turn, can lead to an increased risk of knee injuries [25]. The increased production of sex hormones can trigger significant growth in the long bones of the lower limbs, which precedes neuromuscular and cartilaginous changes. These changes inherent to sexual maturation may result in a higher incidence of knee injuries during puberty [24, 25].

PFPS was positively associated with greater functional impairment. Thus, it is evident that PFPS affects the functionality of adolescents and their social participation [6], and it can also affect their academic performance and quality of life. PFPS is characterized by a condition that can lead to changes in an individual’s functionality, with one of the signs of the syndrome being the presence of pain that limits the performance of certain daily activities [26].

Our results showed no association between posterior chain flexibility and PFPS, corroborating the study by Sannasi, Rajashekar, and Hegde [27], in which no association was observed between this disorder and hamstring tightness in young adults. However, in a recent systematic review with meta-analysis, Alsaleh et al. [28] found moderate evidence that PFPS may be associated with reduced hamstring flexibility in men and women under 40 years old. Despite the controversies, these studies do not provide evidence on this topic for a population of children and adolescents, who certainly present differences in neuromuscular plasticity and development compared to adults. Therefore, the scarcity of studies on this topic, combined with the wide methodological and population variability found in the scientific literature, leaves gaps in understanding the relationship between PFPS and flexibility in these individuals. Hence, studies with different methodological designs that involve children are important.

Furthermore, we observed that overweight/obesity and low weight were not associated with PFPS. These data are supported by other studies that have investigated adolescents with knee pain complaints in a Brazilian population [4], as well as PFPS [29]. It is worth noting that previous studies have found a positive association between overweight/obesity and PFPS and patellar osteoarthritis in adults [29]. At the same time, overweight/obesity in adolescence was associated with knee pain in adulthood [30]. Thus, although obesity/overweight may not seem to be associated with PFPS, the late effects of maintaining high BMI levels cause structural changes in the knee joint in the long term.

This study provides valuable information about PFPS in Brazilian children and adolescents. However, some considerations can be highlighted. The study did not assess participants’ muscle strength and the type of physical activity, which could contribute to a more comprehensive understanding of the factors and biomechanics involved in PFPS mechanisms. Another point to consider is the limitation of the IPAQ as a tool for measuring physical activity levels, as its scores may not be entirely reliable (subject, for example, to response bias). These limitations could somehow interfere with the statistical analysis process.

Despite the considerations, this study brings significant benefits to clinical practice, having valuable aspects for future investigations that aim to better explain the risk factors involved in PFPS in children and adolescents. Identifying a considerable prevalence of PFPS and its associated intrinsic and extrinsic factors in this population can improve clinical diagnosis and treatment provided by healthcare professionals, offering more effective interventions. Additionally, this information can help raise awareness in the community in general about the importance of physical activity from an early age and its significance in promoting healthy habits and preventing long-term musculoskeletal disorders.

A considerable prevalence of PFPS found in children and adolescents of both genders in this study can support the development of public policies for these groups. Thus, actions to prevent the associated factors evaluated in this study, as well as early diagnosis, could minimize the consequences of this painful syndrome. Based on the Brazilian healthcare model [31], primary healthcare services can be powerful tools for screening and managing PFPS in children and students due to their high outreach and impact within communities and schools.

## Supporting information

S1 Table Characterization of study participants (*) number of individuals assessed. (DOCX)

S2 Table Relationship between PFPS and the independent variables of the study. Sample = 283. Prevalence Ratio estimates obtained by Multiple Poisson Regression. Natal/RN, 2020.
(DOCX)

S1 Data set (XLSX)
