# Supplementary material for: Patellofemoral pain syndrome in children and adolescents: A cross-sectional study
Source: PLoS One. 2024 Apr 16;19(4):e0300683. doi: 10.1371/journal.pone.0300683 (PMC11020395; doi:10.1371/journal.pone.0300683)
Supplement: S1 Table — (DOCX) [file pone.0300683.s001.docx]

**S1 Table.** Characterization of study participants (*) Number of individuals assessed.

|  | **Prevalence** | | |
| --- | --- | --- | --- |
| **Variable** | **n (*)** | **% (**)** | **CI (95%)** |
| **Patellofemoral Pain Syndrome (PFPS)** |  |  |  |
| Absent | 210 | 75.3 | 68.6 - 81 |
| Present | 73 | 24.7 | 19 – 31.4 |
| **Sex** |  |  |  |
| Male | 131 | 49.1 | 41.9 – 56.4 |
| Female | 152 | 50.9 | 43.6 – 58.1 |
| **Age Range** |  |  |  |
| 10 to 12 years | 22 | 12.2 | 7.1 – 20.1 |
| 13 to 15 years | 79 | 29.7 | 23.5 – 36.8 |
| 16 to 18 years | 182 | 58.1 | 50.5 – 65.3 |
| **Physical Activity – IPAQ Questionnaire** |  |  |  |
| Irregularly Active | 85 | 29.2 | 23.4 – 35.7 |
| Active | 121 | 45.9 | 38.5 – 53.3 |
| Very Active | 77 | 25.0 | 19.4 – 31.6 |
| **Body Mass Index** |  |  |  |
| Adequate | 176 | 65 | 57.5 – 71.8 |
| Underweight | 37 | 8 | 5.5 – 11.4 |
| Overweight | 68 | 27 | 20.6 – 34.7 |
| **Sexual Maturation** |  |  |  |
| Post-pubertal | 78 | 29.7 | 23.5 – 36.8 |
| Pubertal | 205 | 70.3 | 63.2 – 76.5 |
| **Anterior Knee Pain Scale – Kujala Questionnaire** | | |  |
| No Functional Impairment | 214 | 78.9 | 73.1 – 83.7 |
| Functional Impairment | 69 | 21.1 | 16.3 – 26.9 |
| **Posterior Chain Flexibility** | |  |  |
| Adequate | 120 | 42.3 | 35.2 – 49.7 |
| Inadequate | 163 | 57.7 | 50.3 – 64.8 |

(*) Number of individuals assessed.

(**) Percentage obtained after weighting and cluster effect. Does not correspond to the same sample proportion.
