# Supplementary material for: Patellofemoral pain syndrome in children and adolescents: A cross-sectional study
Source: PLoS One. 2024 Apr 16;19(4):e0300683. doi: 10.1371/journal.pone.0300683 (PMC11020395; doi:10.1371/journal.pone.0300683)
Supplement: S2 Table — Sample = 283. Prevalence Ratio estimates obtained by Multiple Poisson Regression. Natal/RN, 2020. (DOCX) [file pone.0300683.s002.docx]

**S2 Table.** Relationship between PFPS and the independent variables of the study. Sample = 283. Prevalence Ratio estimates obtained by Multiple Poisson Regression. Natal/RN, 2020

|  | **PFPS** | | **Unadjusted** | | **Adjusted** | |
| --- | --- | --- | --- | --- | --- | --- |
|  | Absent  n* (%**) | Present  n* (%**) | p-value | PR (CI95%) | p-value | PR (CI 95%) |
| **Sex** | | | | | | |
| Male | 104 (39.5) | 27 (9.7) |  | 1 |  | 1 |
| Female | 106 (35.9) | 46 (15) | 0.06 | 1.4 (0.9 – 2.2) | 0.1 | 1.3 (0.9 – 2.1) |
| **IPAQ** | | | | | | |
| Irregularly Active | 73 (25) | 12 (4.2) |  | 1 |  |  |
| Active | 78 (30.6) | 43 (15.2) | <0.01 | 2.5 (1.3 – 4.4) | <0.01 | 2.5 (1.4 – 4.5) |
| Very Active | 59 (19.7) | 18 (5.3) | 0.13 | 1.6 (0.8 – 3.2) | 0.19 | 1.6 (0.8 – 3.2) |
| **Sexual Maturation** | | | | | | |
| Post-pubertal | 66 (22.7) | 12 (7) |  | 1 |  | 1 |
| Pubertal | 144 (52.6) | 61 (17.7) | 0.02 | 1.9 (1.1 – 3.3) | 0.03 | 1.8 (1.0 – 3.2) |
| **Posterior Chain Flexibility** | | | | | | |
| Adequate | 93 (31.9) | 27 (10.4) |  | 1 |  |  |
| Inadequate | 117 (43.4) | 46 (14.3) | 0.2 | 1.2 (0.8 – 1.8) | 0.2 | 1.2 (0.8 – 1.9) |
| **Body Mass Index** | | | | | | |
| Adequate | 139 (67.9) | 39 (55.9) |  | 1 |  |  |
| Underweight | 24 (7.1) | 13 (10.9) | 0.08 | 1.5 (0.9 – 2.6) | 0.09 | 1.5 (0.9 – 2.5) |
| Overweight | 49 (25.1) | 19 (33.3) | 0.3 | 1.2 (0.7 – 2.0) | 0.3 | 1.2 (0.7 – 1.9) |
| **Functional Capacity – Kujala Questionnaire** | | | | | | |
| Unaltered | 194 | 20 |  | 1 |  | 1 |
| FI | 16 | 53 | <0.01 | 8.2 (5.3 – 12.2) | <0.01 | 8.0 (5.0 – 12.8) |

IPAQ: International Physical Activity Questionnaire. BMI: Body Mass Index; FI: Functional Impairment.

(*) Number of individuals assessed.

(**) Percentage obtained after weighting and cluster effect. Does not correspond to the same sample proportion.

Sample = 283. Prevalence Ratio estimates obtained by Multiple Poisson Regression. Natal/RN, 2020.
